# Decreased NK cell count is a high-risk factor for convulsion in children with COVID-19

**DOI:** 10.1186/s12879-023-08556-7

**Published:** 2023-12-06

**Authors:** Ting Shi, Yuanxi Bian, Jiahui Wu, Xiaohong Li, Jianping Deng, Tao Feng, Linlin Huang, Xiaoxing Kong, Jianmei Tian

**Affiliations:** Department of Infectious Diseases & Pediatric Intensive Care Unit, Children’s Hospital of Soochow University, 303 Jingde Road, Suzhou, 215000 Jiangsu China

**Keywords:** Convulsion, Children, NK cells, Omicron

## Abstract

**Background:**

The neurological symptoms caused by severe acute respiratory syndrome coronavirus 2 (SARS-CoV-2) are of increasing concern. Convulsions are among the main neurological manifestations reported in children with coronavirus disease-2019 (COVID-19), and cause serious harm to physical and mental health. This study aimed to investigate the risk factors for convulsion in children with COVID-19.

**Methods:**

This prospective study was conducted at the Children’s Hospital of Soochow University. In total, 102 COVID-19 patients with convulsion, 172 COVID-19 patients without convulsion, and 50 healthy controls were enrolled in the study. The children’s clinical and laboratory data were analyzed to assess the risk factors for convulsion in COVID-19 patients.

**Results:**

Convulsions occurred in 37.2% of children, mostly those aged 1–3 years, who were hospitalized with the Omicron variant. The neutrophil count, neutrophil-to-lymphocyte ratio (NLR), monocyte-to-lymphocyte ratio (MLR), platelet-to-lymphocyte ratio (PLR), and mean platelet volume-to-platelet ratio (MPR) were significantly higher in the convulsion group than those in the non-convulsion and control groups (*P* < 0.01). However, the counts of lymphocytes, eosinophils, platelets, lymphocyte subsets, CD3^+^ T cells, CD4^+^ T cells, CD8^+^ T cells, and NK cells were lower in the convulsion group than those in the non-convulsion and control groups (*P* < 0.01). Multivariate regression analysis indicated that NK cell count (OR = 0.081, 95% CI: 0.010–0.652) and a history of febrile seizure (OR = 10.359, 95% CI: 2.115–50.746) were independent risk factors for the appearance of convulsions in COVID-19.

**Conclusions:**

History of febrile seizure and decreased NK cell count were high-risk factors for convulsions in COVID-19 patients.

**Supplementary Information:**

The online version contains supplementary material available at 10.1186/s12879-023-08556-7.

## Background

Coronavirus disease-2019 (COVID-19), caused by severe acute respiratory syndrome coronavirus 2 (SARS-CoV-2), has had a tremendous influence on human society. As of January 1, 2023, COVID-19 has resulted in over 656 million confirmed cases and 6.6 million deaths worldwide, according to the World Health Organization [1]. Since November 2021, SARS-CoV-2 Omicron subvariants, such as BA1, BA2, BA4, BA5, BF. 7, BQ. 1, BQ. 1, and the recombinant variant XBB have appeared worldwide and gradually become the predominant circulating strains [1]. The subvariants of Omicron are characterized by strong infectivity, short latency, and immune escape. According to the National Center for Disease Control and Prevention (CDC) in China, the prevalent strains during our study were BA. 5.2 and BF. 7 [2].

SARS-CoV-2 infection has generated a variety of clinical symptoms. In addition to the ubiquitous respiratory symptoms, some children have also exhibited neurological symptoms during the Omicron wave [3]. A multi-center cohort study in the United States demonstrated that 40% of children with COVID-19 exhibited at least one neurological symptom [4]. A systematic review by Misra et al. indicated that up to a third of COVID-19 inpatients experienced neurological symptoms [5]. LaRovere et al. reported that 22% of children with COVID-19 had a neurological involvement [6]. The neurological injuries caused by COVID-19 in children were mainly manifested as headache, myalgia, anosmia, febrile seizures (FSs), encephalitis, myelitis, meningitis, encephalopathy, Guillain-Barré syndrome, and acute disseminated encephalomyelitis, among which anosmia and febrile seizures were the most common [6]. FSs are increasingly recognized by physicians as a pediatric emergency and early manifestation of acute necrotizing encephalopathy, encephalitis, and meningitis. Serious nervous system involvement, such as severe encephalopathy or brain edema, was observed in 2.5% of children infected with Omicron subvariants [7]. The mortality rate among these patients was as high as 25.5% [6]. Persistent and recurrent seizures were the main early manifestations of necrotizing encephalopathy and brain edema. If the occurrence of acute neurological damage can be predicted early, this can facilitate timely intervention for children and improve their prognosis.

SARS-CoV-2 is transmitted by direct viral spread and through droplets/airborne from infected patients. It invades vascular, airway, and alveolar epithelial cells, endothelial cells, and macrophages by attaching to the ACE2 receptor [8]. An uncontrolled innate immune response such as the excessive release of interleukin-6 (IL-6), IFN-γ, and monocyte chemoattractant protein-1, and imbalanced adaptive immunity are two ways in which SARS-CoV-2 induces lung tissue damage [9]. In addition, it is currently believed that the pathogenic mechanism of SARS-CoV-2 infection on the central nervous system (CNS) involves direct invasion of the CNS [10] and an excessive release of pro-inflammatory cytokines, such as tumor necrosis factor-α and interleukins (IL) -1β, -6, -8 and -17 [11, 12]. Although several review articles have described the respiratory and neurological manifestations of COVID-19 [13, 14], the demographic characteristics of presentations involving different organ systems after SARS-CoV-2 infection have not been explored. This study intended to compare clinical and experimental indicators of SARS-CoV-2 infection in children presenting with respiratory and neurological involvement. The secondary objective was to investigate high-risk factors for the development of convulsions in children with COVID-19 to provide an early warning and clues to their pathogenesis.

## Results

### Clinical characteristics of the convulsion and non-convulsion groups in inpatients infected by the Omicron variant

The study cohort had a total of 274 inpatients infected by the Omicron variant, with 102 patients experiencing convulsion and 172 patients without convulsion. There were 61 males and 41 females, with a median age of 2.1 (1.4–3.4) years in the convulsion group, and 97 males and 75 females, with a median age of 1.1 (0.25–5.1) years in the non-convulsion group (Table 1). There was no significant difference in the sex ratio between the two groups. A greater proportion of children in the convulsion group were aged 1–3 years and were older compared with the children in the non-convulsion group (*P* < 0.05). In addition, children in the convulsion group were more likely to have a history of febrile seizure (FS) than those in the non-convulsion group. The duration in the convulsion group was shorter than that in the non-convulsion group (*P* < 0.05). However, patients in the non-convulsion group were more likely to exhibit cough, wheeze, and polypnea than those in the convulsion group (*P* < 0.05).

**Table 1:** General characteristics of inpatients infected by Omicron variant with and without convulsion

Parameters	Convulsion group (*n* = 102)	Non-convulsion group (*n* = 172)	*P*
Sex(male)	61(59.8)	97(56.3)	0.581
Age(years)	2.1(1.4–3.4)	1.1(0.25–5.1)	0.01
Age group			
≤ 1 year	11(10.8)	83(48.3)	< 0.01
1- ≤ 3 years	60(58.8)	27(15.7)	< 0.01
3- ≤ 6 years	16(15.7)	28(16.3)	0.897
> 6 years	15(14.7)	34(19,8)	0.291
Disease course(days)	1(1–2)	4(2–6.7)	< 0.01
Fever	100(98.0)	164(95.3)	0.251
Peak of fever (℃)	39.4 ± 0.7℃	39.1 ± 1.1	0.0734
History of febrile seizure	33(32.4)	6(3.5)	< 0.01
Oxygen saturation (on admission, %)	97.7 ± 1.3	98.0 ± 1.2	0.141
Cough	75(73.5)	155(90.1)	< 0.01
Wheeze	1(1.0)	11(6.4)	0.034
Polypnea	3(2.9)	17(9.9)	0.033
Hoarse	9(8.8)	23(13.4)	0.257
Vomit	15(14.7)	24(13.9)	0.863
Diarrhea	7(6.9)	1(0.6)	0.003
Rash	0(0.0)	6(3.5)	0.087

The data are reported as median (interquartile range), mean ± standard deviation or n (%). The univariate analyses were performed using Mann–Whitney U test for skewed distributed data, T-test for normally distributed data and the chi-square test or fisher′s exact test for categorical variables. *P* < 0.05 had statistical significance

### Laboratory parameters of the convulsion, non-convulsion, and control groups

As shown in Tables 2 and 3, there were no significant differences in sex ratio and age between the control and case groups (*P* > 0.05). The neutrophil count, neutrophil-to-lymphocyte ratio (NLR), monocyte-to-lymphocyte ratio (MLR), platelet-to-lymphocyte ratio (PLR), and mean platelet volume-to-platelet ratio (MPR) were significantly higher in the convulsion group than those in the non-convulsion and control groups (*P* < 0.01). However, the lymphocyte count, eosinophil count, platelet, lymphocyte subsets, CD3^+^ T cell count, CD4^+^ T cell count, CD8^+^ T cell count, and NK cell count were lower in the convulsion group than those in the non-convulsion and control groups (*P* < 0.01). In addition, the monocyte count and the globulin, ALT, AST, LDH, and C_4_ values were higher in the convulsion and non-convulsion groups than those in the control group (*P* < 0.01). The ALP value and CD3^−^CD19^+^ B cell count were lower in the convulsion and non-convulsion groups than those in the control group (*P* < 0.01). The value of procalcitonin (PCT) was higher in the convulsion group compared to the non-convulsion group (*P* < 0.01). There were no significant differences in IgA, IgG, or IgM among the three groups (*P* > 0.05). The majority of children in both the convulsion and non-convulsion groups had decreased serum calcium and increased lactate and D-Dimer. The proportion of children with elevated lactate was higher in the non-convulsion group than in the convulsion group (*P* < 0.01).

**Table 2:** The hematological profiles of COVID-19 patients infected by Omicron variant with and without convulsion

Parameters	Convulsion group (*n* = 102)	Non-convulsion group (*n* = 172)	Control group (*n* = 50)	*P*
Sex(male)	61(59.8)	97(56.3)	24(48.0)	0.577
Age(years)	2.1(1.4–3.4)a	1.1(0.25–5.1)b	2.5(0.8–4.0)ab	0.04
WBC (× 10^9^/L)	7.9(5.3–9.5)	7.2(5.2–10.1)	8.1(6.9–9.8)	0.128
Neutrophils, (× 10^9^/L)	4.7(2.4–6.4)a	2.9(1.6–5.1)b	2.6(1.7–3.6)b	< 0.01
Lymphocytes, (× 10^9^/L)	1.6(0.9–2.5)a	2.5(1.6–4.3)b	4.7(3.3–5.5)c	< 0.01
Monocytes, (× 10^9^/L)	0.7(0.5–1.0)a	0.7(0.5–1.0)a	0.5(0.3–0.7)b	< 0.01
Eosinophils, (× 10^9^/L)	0.02(0.01–0.08)a	0.07(0.01–0.16)b	0.1(0.1–0.2)c	< 0.01
NLR	3.0(0.9–5.4)a	1.2(0.4–2.4)b	0.5(0.3–0.8)c	< 0.01
MLR	0.4(0.2–0.7)a	0.2(0.1–0.4)b	0.1(0.08–0.1)c	< 0.01
Hemoglobin, (g/L)	117.0(114.0–124.0)	118.0(109.0–128.0)	120.0(114.0–125.0)	0.799
Platelet, (× 10^9^/L)	232.0 ± 76.5a	284 ± 115.7b	300 ± 75b	< 0.01
MPV,fL	9.5 ± 1.2	9.7 ± 1.2	9.6 ± 0.8	0.335
PLR	132.4(83.0–231.2)a	109.3(66.6–167.0)b	66.1(53.5–82.0)c	< 0.01
MPR	0.04(0.03–0.05)a	0.03(0.02–0.04)b	0.03(0.02–0.03)b	< 0.01
CRP (> 8mg/L)	30(29.4)	55(31.9)	[0.0–8.0]	0.657
PCT (> 0.5ng/mL)	31(30.4)a	16(9.3)b	[≤ 0.5]	< 0.01

The data presented as median (interquartile range), mean ± standard deviation, [reference value] and n(%). The univariate analyses were performed using Kruskal–Wallis for skewed distribution variables, ANOVA for normal distribution variables and the chi-square test for categorical variables. Abbreviation: *WBC* white blood cell, *NLR* neutrophil-to-lymphocyte ratio, *MLR* monocyte-to-lymphocyte ratio, *MPV* mean platelet volume, *PLR* platelet-to-lymphocyte ratio, *MPR* mean platelet volume-to-platelet ratio, *CRP* C-reactive protein, *PCT* procalcitonin. *P* < 0.05 between a, b and c

**Table 3:** The biochemical and lymphocyte subsets examination of COVID-19 patients infected by Omicron variant with and without convulsion

Parameters	Convulsion group (*n* = 102)	Non-convulsion group (*n* = 172)	Control group (*n* = 50)	*P*
Albumin(g/L)	44.2 ± 2.2a	42.6 ± 4.1b	44.9 ± 2.7a	< 0.01
Globulin(g/L)	23.1(21.0–25.2)a	23.2(19.5–27.2)a	21.2(19.0–23.6)b	0.027
ALT(U/L)	20.5(15.8–25.4)a	22.9(14.6–42.1)a	15.5(13.1–18.3)b	< 0.01
AST(U/L)	51.3(42.5–63.8)a	51.8(36.7–72.5)a	34.6(28.7–38.8)b	< 0.01
ALP(U/L)	224.9 ± 60.9a	205.2 ± 78.9b	258.9 ± 79.5c	< 0.01
LDH(U/L)	345.0(304.0–419.0)a	372.4(308.5–449.8)a	261(242–291)b	< 0.01
C3(g/L)	1.0(0.9–1.2)	1.1(0.8–1.3)	1.0(0.9–1.1)	0.952
C4(g/L)	0.3(0.3–0.4)a	0.3(0.2–0.4)a	0.2(0.2–0.3)b	< 0.01
IgA(g/L)	0.4(0.2–0.8)	0.3(0.1–1.2)	0.6(0.2–1.0)	0.226
IgG(g/L)	6.7(5.6–8.3)	6.5(4.1–8.9)	7.0(5.9–9.4)	0.080
IgM(g/L)	1.0(0.7–1.3)	0.8(0.5–1.3)	0.9(0.6–1.4)	0.050
Ca2 + (< 1.12mmol/L)	77(75.5)	113(65.7)	[1.12–1.27]	0.089
Lactic acid (> 1.7mmol/L)	55(53.9)	133(77.3)	[0.5–1.7]	< 0.01
D- Dimer (> 550ug/L)	46(45.1)	87(50.6)	[0–550]	0.380
lymphocyte subsets Log(/ul)	3.2 ± 0.3a	3.4 ± 0.3b	3.6 ± 0.2c	< 0.01
CD3^+^ count Log(/ul)	3.0 ± 0.4a	3.2 ± 0.4b	3.4 ± 0.2c	< 0.01
CD3^+^CD4^+^ count Log(/ul)	2.7 ± 0.4a	3.0 ± 0.4b	3.2 ± 0.2c	< 0.01
CD3^+^CD8^+^ count Log(/ul)	2.5 ± 0.4a	2.7 ± 0.4b	3.0 ± 0.2c	< 0.01
CD3^−^CD19^+^count Log(/ul)	2.6 ± 0.4a	2.7 ± 0.4a	3.0 ± 0.2b	< 0.01
NK count Log(/ul)	2.2 ± 0.4a	2.4 ± 0.4b	2.5 ± 0.3b	< 0.01

The data presented as median (interquartile range), mean ± standard deviation, [reference value] and n (%). The univariate analyses were performed using Kruskal–Wallis for skewed distribution variables, ANOVA for normal distribution variables and the chi-square test for categorical variables

*Abbreviation:* *ALT* Alanine transaminase, *AST* Aspartate transaminase, *ALP* Alkaline phosphatase, *LDH* Lactate dehydrogenase

*P* < 0.05 between a, b and c

### The risk factors for convulsion in children with SARS-CoV-2 infection

**Fig. 1:**
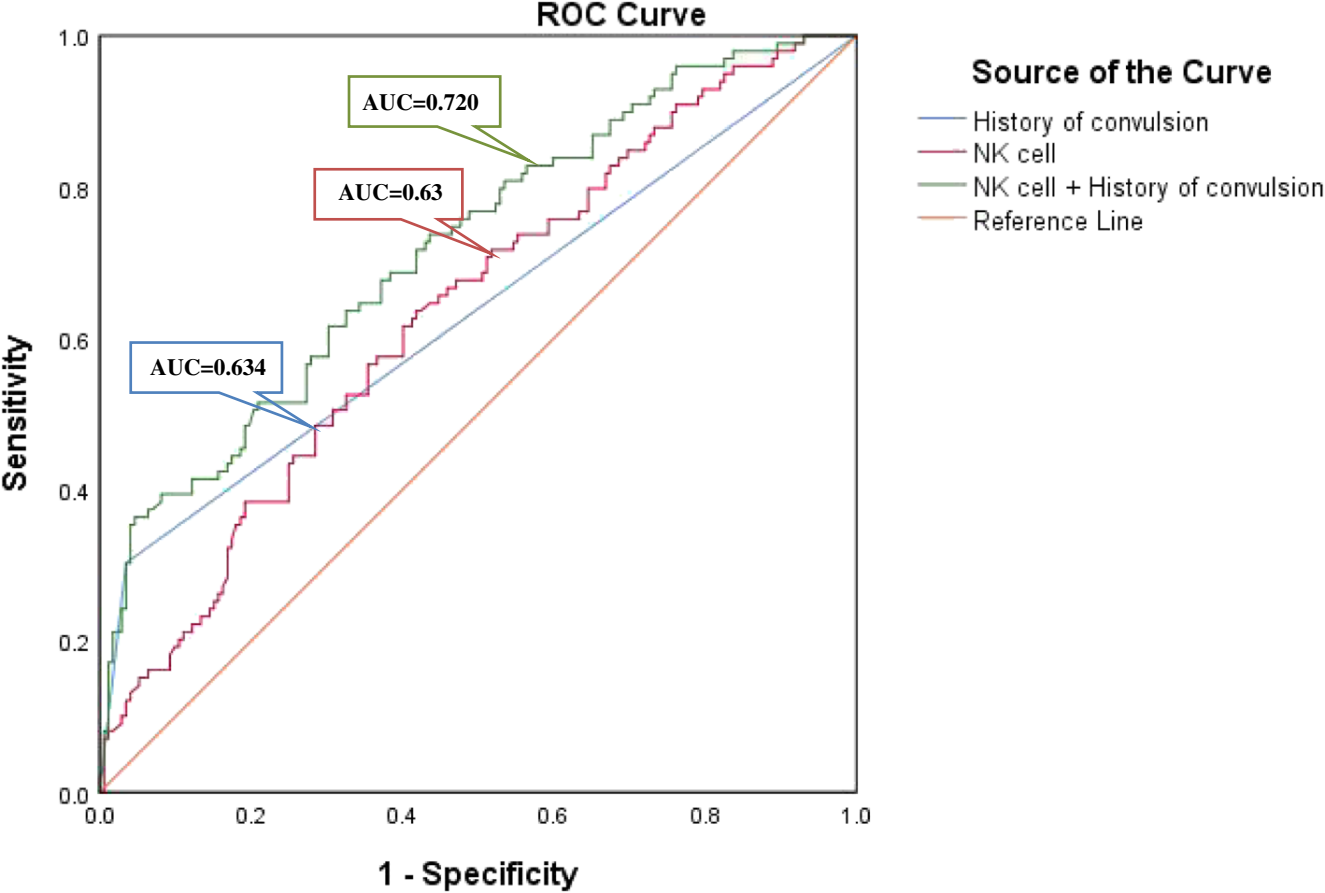
Diagnostic value of each indicator for predicting convulsions caused by Omicron in children

The clinical and laboratory parameters with statistically significant differences between the convulsion and non-convulsion groups were included in the logistic regression analysis. The three models were built for regression analysis (Table 4). In Model 1 (without any correction factors), history of febrile seizure, cough, polypnea, neutrophils, lymphocytes, eosinophils, NLR, MLR, platelet, PLR, albumin, ALP, lymphocyte subsets, CD3 + T cell count, CD3 + CD4 + T cell count, CD3 + CD8 + T cell count and NK cell count were statistically significant indices for predicting convulsion (*P* < 0.05). After correcting for gender and age in Model 2, eosinophils and albumin were no longer statistically significant. All indicators were adjusted in Model 3 and history of febrile seizure and NK cell count were independent risk factors for convulsions in children with SARS-CoV-2 infection. In addition, ROC curve analysis indicated that the diagnostic value of history of febrile seizure combined with NK cell count was 0.720 (95% CI: 0.657–0.783,* P* < 0.01; Fig. 1 and Table 5).

**Table 4:** Factors associated with convulsion in COVID-19 patients infected by Omicron variant (multivariate analysis)

Variable	Model 1		Model 2		Model 3	
	OR (95%CI)	*P*	OR (95%CI)	*P*	OR (95%CI)	*P*
History of febrile seizure	12.029(4.792–30.196)	< 0.01	10.970(3.986–30.192)	< 0.01	10.359(2.115–50.746)	0.004
Cough	0.305(0.156–0.593)	< 0.01	0.260(0.117–0.576)	0.001	0.816(0.242–2.752)	0.743
Wheeze	0.145(0.018–1.139)	0.066				
Polypnea	0.276(0.079–0.967)	0.044	0.179(0.045–0.712)	0.015	0.153(0.022–1.081)	0.060
Diarrhea	0.454(0.188–1.096)	0.079				
Neutrophils(× 10^9^/L)	1.123(1.035–1.217)	0.005	1.116(1.015–1.226)	0.023	1.033(0.765–1.395)	0.831
Lymphocytes(× 10^9^/L)	0.712(0.605–0.837)	< 0.01	0.756(0.621–0.920)	0.005	1.335(0.894–1.996)	0.158
Eosinophils(× 10^9^/L)	0.012(0.001–0.184)	0.001	0.139(0.013–1.476)	0.102		
NLR	1.261(1.135–1.401)	< 0.01	1.213(1.084–1.359)	0.001	1.055(0.730–1.525)	0.774
MLR	7.815(3.202–19.075)	< 0.01	8.464(3.033–23.616)	< 0.01	0.769(0.087–6.760)	0.813
Platelet(× 10^9^/L)	0.995(0.992–0.997)	< 0.01	0.997(0.994–1.000)	0.047	0.996(0.988–1.005)	0.412
PLR	1.005(1.002–1.008)	< 0.01	1.005(1.002–1.008)	< 0.01	1.003(0.991–1.015)	0.680
MPR	5.151(0.009–3022.779)	0.614				
PCT(ng/mL)	1.018(0.960–1.078)	0.556				
Albumin(g/L)	1.241(1.114–1.382)	< 0.01	1.127(0.994–1.277)	0.062		
ALP(U/L)	1.004(1.000–1.007)	0.041	1.010(1.005–1.015)	< 0.01	1.001(0.994–1.008)	0.712
lymphocyte subsets Log(/ul)	0.172(0.078–0.377)	< 0.01	0.138(0.048–0.394)	< 0.01	9.525(0.112–54.807)	0.687
CD3^+^T cell count Log(/ul)	0.179(0.087–0.371)	< 0.01	0.185(0.074–0.464)	< 0.01	0.62(0.120–16.368)	0.779
CD3^+^CD4^+^T cell count Log(/ul)	0.198(0.103–0.383)	< 0.01	0.187(0.078–0.449)	< 0.01	0.291(0.112–11.38)	0.819
CD3^+^CD8^+^T cell count Log(/ul)	0.237(0.117–0.480)	< 0.01	0.226(0.097–0.529)	0.001	21.994(0.220–21.541)	0.379
CD3^−^CD19^+^ B cell count Log(/ul)	0.552(0.288–1.059)	0.074				
NK count Log(/ul)	0.255(0.127–0.512)	< 0.01	0.254(0.109–0.595)	0.002	0.081(0.010–0.652)	0.018

*Abbreviations:* *NLR* neutrophil-to-lymphocyte ratio, *MLR* monocyte-to-lymphocyte ratio, *PLR* platelet-to-lymphocyte ratio, *MPR* mean platelet volume-to-platelet ratio, *PCT* procalcitonin, *ALP* alkaline phosphatase, *OR* odds ratio, *CI* confidence interval. Model 1 was not adjusted. Model 2 was adjusted for age and sex. Model 3 was adjusted as Model 2 + disease course, history of convulsion, cough, polypnea, neutrophils, lymphocytes, NLR, MLR, platelet, PLR,ALP, lymphocyte, CD3^+^ T cell count, CD3^+^CD4^+^ T cell count, CD3^+^CD8^+^ T cell count and NK cell count

**Table 5:** AUC of each indicator for predicting convulsions in children with Omicron infection

Variable	AUC	95%CI	*P*
History of convulsion	0.634	0.562–0.706	< 0.01
NK cell	0.636	0.568–0.703	< 0.01
NK cell + History of convulsion	0.720	0.657–0.783	< 0.01

*Abbreviations:* *AUC* area under curve, *CI* confidence interval

### Clinical characteristics of convulsion groups I and II

Convulsions induced by SARS-CoV-2 infection manifested either as a single convulsion, multiple convulsions, or status epileptic. In this study, the children with a single convulsion and convulsion time of < 5 min were classified as group I and those with multiple convulsions or status epileptic were classified as group II. As shown in Table S1 and S2, there was no significant difference between groups I and II in clinical manifestations or blood routine indexes. However, globulin and IgA were lower in group II than in group I, and ALP was higher in group II than in group I (*P* < 0.05, Table S3).

## Discussion

Since the COVID-19 epidemic began, the neurological symptoms it caused have become increasingly recognized by physicians. It was important to ascertain the clinical presentation and laboratory features of SARS-CoV-2-associated neurological symptoms, as nervous system inflammation could result in significant morbidity and mortality [15]. In this study, 37.2% (102/274) of the children with COVID-19 had convulsions, which is consistent with the findings of previous studies by Fink et al. (40%) [4] and Lin et al. (43%) [16]. A greater proportion of COVID-19 patients with convulsions were aged 1–3 years and were older than those without convulsions, which was also consistent with results reported in a previous study [4]. In this study, 98.0% of COVID-19 patients with convulsions had fever and were diagnosed with febrile seizures (FSs), which illustrated that FS was the most common neurological sign of SARS-CoV-2 infection [17]. In addition, our findings indicated that children with COVID-19 who had a history of FS were more likely to have convulsions than those without a history of FS, independent of the febrile peak of the child's fever.

Hematological parameters play a significant role in the early diagnosis of multiple inflammatory illnesses [18–20]. In this study, COVID-19 patients had lower counts of lymphocytes, eosinophils, and platelets and higher monocyte counts compared with those in healthy children, which is consistent with the results of previous studies [21, 22]. The lymphopenia was associated with viral infection of lymphocytes via angiotensin-converting enzyme 2 (ACE2) receptors, leading to lymphocyte apoptosis [23]. Contrary to previous research [24], neutrophils were elevated rather than decreased in COVID-19 patients in this study, particularly in those with convulsions. This may be related to granulocyte irregularities in severe COVID-19 as well as infection with different viral variant strains [25]. The neutrophil-to-lymphocyte ratio (NLR), monocyte-to-lymphocyte ratio (MLR), and platelet-to-lymphocyte ratio (PLR) can also be indicators of early inflammation and have been associated with the severity of COVID-19 [26, 27]. However, linkages of these parameters to SARS-CoV-2-related nervous system injury have been limited. COVID-19 patients with elevated NLR, MLR, and PLR were described in this study, and elevations of these parameters were more pronounced in patients with convulsions. This implies that the inflammatory response was stronger in COVID-19 patients with convulsions than in those without convulsions.

We also analyzed the peripheral blood lymphocyte subsets of children infected with SARS-CoV-2. In addition to the decreased total lymphocyte count, the subsets of CD4 ^+^ T cells, CD8 ^+^ T cells, CD19^+^B cells, and NK cells were also decreased. SARS-CoV-2 infection is fought by cytotoxic CD8^+^ T cells, CD4^+^T helper cells, NK cells, and B cells [25]. CD8^+^T cells and NK cells kill virus-infected cells, and B cells emit neutralizing antibodies [28]. However, SARS-CoV-2 can disrupt normal immune responses by depleting the immune process and producing an uncontrolled inflammatory response [25, 29]. Multivariate logistic regression analysis revealed that a history of FS and a decreased NK cell count were independent risk factors for the development of convulsions in children with SARS-CoV-2 infection. Our results illustrate that NK cells played an important role in the occurrence of convulsions in COVID-19 patients. However, IgA, IgM, and IgG were not elevated in the children with SARS-CoV-2 infection in this study, which may be related to the strong immune escape effect of the virus.

It is well known that SARS-CoV-2 infection in children causes multiple organ injury. Mild elevations of ALT and AST were present in this study, which was consistent with previous studies [30]. Furthermore, SARS-CoV-2 infection-induced liver failure has been previously reported [31]. Interestingly, the values of serum ALP and Ca^2+^ decreased in COVID-19 patients in this study. A previous study found that vitamin D was closely associated with the severity of COVID-19 [32]. Therefore, we speculate that SARS-CoV-2 might interfere with calcium metabolism and that this may be involved in the occurrence of convulsions.

The current study concluded that patients with SE and multiple convulsions were more likely to progress to severe encephalitis, meningitis, and encephalopathy [33]. In the present study, there was no significant difference in clinical and peripheral blood indices between patients with a single convulsion and those with multiple convulsions. It is noteworthy that IgA was at low levels in children with complex convulsions. The results of a previous study [34] also indicated that plasma IgA level was associated with prognosis and illustrated that IgA played a protective role in controlling SARS-CoV-2 infection.

## Conclusion

In this study, 37.2% of children hospitalized with the Omicron variant had convulsions, which mostly occurred in children 1–3 years of age. Decreased lymphocytes and eosinophils and elevated monocytes were hematological characteristics of SARS-CoV-2 infection in children. In addition, decreased NK cell count and history of FS were independent risk factors for the appearance of convulsions in children with COVID-19. Finally, our research showed that COVID-19 patients with multiple convulsions and SE had lower IgA levels than those with a single convulsion.

## Methods

### Patient characteristics

At the start of the third week of December 2022, the Omicron variant of SARS-CoV-2 was widely prevalent due to the adjustment of the zero-covid policy in China. This prospective study was conducted at the Children’s Hospital of Soochow University between December 17, 2022 and January 7, 2023. Patients were included in the case group based on the following two criteria: (1) hospitalized patient with positive SARS-CoV-2 detected by polymerase chain reaction (PCR) of a nasopharyngeal swab; (2) an absence of chronic diseases (including metabolic diseases andepilepsy), hematological diseases, or autoimmune diseases, and taking no antiviral drugs. The control group consisted of healthy children who underwent surgery (inguinal hernia and phimosis) and were unaffected for the last 2 weeks. Routine blood tests, C-reactive protein (CRP), procalcitonin (PCT), electrolyte, D-dimer, lymphocyte subsets, complement, immunoglobulin, and SARS-CoV-2 DNA-PCR assays were performed within 24 h of admission. A total of 324 participants (182 males and 142 females; age range 0.7–15 years) were enrolled in this study. The case group included 172 COVID-19 patients without convulsion (97 males and 75 females; age quartile: 0.25–5.1 years), 62 COVID-19 patients with a single convulsion (40 males and 22 females; age quartile: 1.3–4.6 years), 39 COVID-19 patients with multiple convulsions or status epileptic (SE) (21 males and 18 females; age quartile: 1.1–2.7 years). The control group consisted of 50 healthy children (24 males and 26 females; age quartile: 0.8–4.0 years).

This study was approved by the Ethics Committee of the Children’s Hospital of Soochow University, China (No.2019KS004). All participants provided written informed consent.

### Definitions

Status epileptic (SE): either a single unremitting seizure lasting longer than 5 min or frequent clinical episodes without an interictal return to the baseline clinical state [35].

### Laboratory tests

#### SARS-CoV-2 DNA-PCR assay

Nasopharyngeal swabs were collected from all patients during admission for SARS-COV-2 assay. The detection was performed by RT-PCR with the SARS-CoV-2 nucleic acid detection kit (DaAn Gene Co., Ltd). All steps were performed according to the manufacturer's instructions. A value below 5 × 10^2^ copies ml^−1^ was considered negative.

### Routine complete blood count, liver function, and immunoglobulin assays

Routine blood count was conducted using a type BC-5310 instrument (Shenzhen Mindray Biomedical Electronics Co., Ltd). Serum ALT (alanine transaminase), AST (aspartate transaminase), ALP (alkaline phosphatase), and LDH (lactate dehydrogenase) were measured using a lactate dehydrogenase assay. Albumin and globulin were measured by biuret and salt out assays, respectively. These biochemical indicators were detected using a HITACHI 7180 biomedical analyzer. Complement C3, C4, immunoglobulin G (IgG), M (IgM), and A (IgA) were detected using a turbidimetric inhibition immunoassay (Orion Diagnostica Oy).

### Lymphocyte subsets analysis

Lymphocyte subsets including T cells (CD3^+^), helper T cells (CD3^+^CD4^+^), killer T cells (CD3^+^CD8^+^), B cells (CD3^−^CD19^+^), and natural killer cells (CD3^−^CD (16^+^56)^+^) were detected using flow cytometry. Peripheral blood samples were labeled with antibodies, including anti-CD3-fluorescein isothiocyanate, anti-CD45-peridin chlorophyll alpha protein-cyanin 5.5, anti-CD4-phycoerythrin cyanin 7, anti-CD8-allophycocyanin-cyanin 7, anti-CD19-APC and anti-CD16^+^56^−^ phycoerythrin. Each sample was analyzed using a multi-color flow cytometer (BD FACSCanto II).

All steps were performed according to the manufacturer's instructions.

### Statistical analysis

The data were presented as median [interquartile range], mean ± standard deviation, and n (%). The t-test and analysis of variance (ANOVA) were used for normally distributed variables. The Mann–Whitney U and Kruskal–Wallis tests were used for skewed distribution variables. Categorical variables were compared using chi-squared or Fisher’s exact tests. Binary logistic regression analysis was used to calculate the odds ratios (ORs) of variables. Receiver-operating characteristic (ROC) curve analysis was used to evaluate the diagnostic accuracy. The statistical analyses were performed using SPSS version 25.0 (IBM Corp., Armonk, NY, USA). Differences were considered statistically significant at* P*-values < 0.05.

### Supplementary Information

**Additional file 1:** **S1 Table.** General characteristics of COVID-19 patients infected by Omicron variant with single and multiple convulsion.

**Additional file 2:** **S2 Table.** The hematological profiles of COVID-19 patients infected by Omicron variant with convulsion.

**Additional file 3:** **S3 Table.** The biochemical and lymphocyte subsets examination of COVID-19 patients infected by Omicron variant with convulsion.

## Data Availability

The data used in this study are available from the corresponding author on reasonable request.

## Abbreviations

CDC: Disease Control and Prevention
FSs: Febrile seizures
CNS: Central nervous system
PCR: Polymerase chain reaction
CRP: C-reactive protein
PCT: Procalcitonin
SE: Status epileptic
ALT: Alanine transaminase
AST: Aspartate transaminase
ALP: Alkaline phosphatase
LDH: Lactate dehydrogenase
ALT: Alanine aminotransferase
IgG: Immunoglobulin G
IgM: Immunoglobulin G
IgA: Immunoglobulin A
NLR: Neutrophil-to-lymphocyte ratio
MLR: Monocyte-to-lymphocyte ratio
PLR: Platelet-to-lymphocyte ratio
MPR: Mean platelet volume-to-platelet ratio
OR: Odds ratio
CI: Confidence interval
ROC: Receiver operating characteristic

## Declarations

A statement to confirm that all methods were carried out in accordance with relevant guidelines and regulations.
