# Supplementary material for: Decreased NK cell count is a high-risk factor for convulsion in children with COVID-19
Source: BMC Infect Dis. 2023 Dec 6;23:856. doi: 10.1186/s12879-023-08556-7 (PMC10698890; doi:10.1186/s12879-023-08556-7)
Supplement: Supplementary file 1 — Additional file 1: S1 Table. General characteristics of COVID-19 patients infected by Omicron variant with single and multiple convulsion. [file 12879_2023_8556_MOESM1_ESM.doc]

**S1 Table** General characteristics of COVID-19 patients infected by Omicron variant with single and multiple convulsion

| Parameters | Convulsion I  group(n=63) | Convulsion II  group(n=39) | *P* |
| --- | --- | --- | --- |
| Sex(male) | 40(63.5) | 21(53.8) | 0.334 |
| Age(years) | 2.1(1.3-4.6) | 1.9(1.1-2.7) | 0.148 |
| Disease course(days) | 1.5±0.8 | 1.5±1.0 | 0.866 |
| Fever | 62(98.4) | 38(97.4) | 0.730 |
| Peak of fever (℃) | 39.4±0.7 | 39.6±0.6 | 0.170 |
| history of convulsion | 17(27.0) | 16(41.0) | 0.141 |
| Oxygen saturation  (on admission, %) | 97.7±1.4 | 97.7±1.5 | 0.847 |
| Cough | 47(74.6) | 28(71.7) | 0.755 |
| Wheeze | 1(1.5) | 0(0.0) | 1.000 |
| Polypnea | 2(3.2) | 1(2.5) | 1.000 |
| Hoarse | 4(6.3) | 5(12.8) | 0.297 |
| Vomit | 11(17.4) | 4(10.3) | 0.318 |
| Diarrhea | 4(6.3) | 3(7.7) | 1.000 |
| Rash | 0(0.0) | 0(0.0) | 1.000 |

Notes: The data are reported as median (interquartile range), mean ± standard deviation or n (%). The univariate analyses were performed using Mann-Whitney U test for skewed distributed data, T-test for normally distributed data and the chi-square test or fisher′s exact test for categorical variables. *P*<0.05 had statistical significance.Convulsion I group represented single convulsion and convulsion II group represented multiple convulsion or SE.
