# Supplementary material for: Decreased NK cell count is a high-risk factor for convulsion in children with COVID-19
Source: BMC Infect Dis. 2023 Dec 6;23:856. doi: 10.1186/s12879-023-08556-7 (PMC10698890; doi:10.1186/s12879-023-08556-7)
Supplement: Supplementary file 2 — Additional file 2: S2 Table. The hematological profiles of COVID-19 patients infected by Omicron variant with convulsion. [file 12879_2023_8556_MOESM2_ESM.doc]

**S2 Table** The hematological profiles of COVID-19 patients infected by Omicron variant with convulsion

| Parameters | Convulsion I group  (n=63) | Convulsion II group  (n=39) | *P* |
| --- | --- | --- | --- |
| WBC  (×10^9^/L) | 7.9±3.4 | 7.4±2.8 | 0.797 |
| Neutrophils, (×10^9^/L) | 4.6(2.4-6.4) | 4.9(2.1-6.2) | 0.813 |
| Lymphocytes, (×10^9^/L) | 1.6(1.0-2.6) | 1.6(0.9-2.5) | 0.669 |
| Monocytes,  (×10^9^/L) | 0.7±0.3 | 0.8±0.3 | 0.181 |
| Eosinophils,  (×10^9^/L) | 0.02(0.01-0.09) | 0.02(0.01-0.06) | 0.994 |
| NLR | 2.8(0.8-4.8) | 3.3(1.0-5.7) | 0.971 |
| MLR | 0.4(0.2-0.7) | 0.5(0.2-0.7) | 0.216 |
| Hemoglobin,  (g/L) | 118±8.5 | 118.7±6.9 | 0.772 |
| Platelet,  (×109/L) | 231±80.4 | 232.2±70.6 | 0.984 |
| MPV, fL | 9.4±1.1 | 9.5±1.1 | 0.661 |
| PLR | 130.7(83.6-221.1) | 154.3(70.4-245.8) | 0.703 |
| MPR | 0.04(0.03-0.05) | 0.04(0.03-0.05) | 0.837 |
| CRP, mg/L | 4.1(1.4-17.5) | 2.7(1.3-7.5) | 0.302 |
| PCT，ng/L | 0.3(0.1-0.6) | 0.3(0.1-1.0) | 0.594 |

Notes: The data presented as median [interquartile range], mean± standard deviation and n (%). The univariate analyses were performed using Mann-Whitney U-test for skewed distribution variables, t-test for normal distribution variables and the chi-square test for categorical variables. Abbreviation: WBC: white blood cell, NLR: neutrophil-to-lymphocyte ratio, MLR: monocyte-to-lymphocyte ratio, MPV: mean platelet volume, PLR: platelet-to-lymphocyte ratio, MPR:mean platelet volume-to-platelet ratio, CRP:C-reactive protein, PCT: procalcitonin. *P*<0.05 had statistical significance.
