# Supplementary material for: Decreased NK cell count is a high-risk factor for convulsion in children with COVID-19
Source: BMC Infect Dis. 2023 Dec 6;23:856. doi: 10.1186/s12879-023-08556-7 (PMC10698890; doi:10.1186/s12879-023-08556-7)
Supplement: Supplementary file 3 — Additional file 3: S3 Table. The biochemical and lymphocyte subsets examination of COVID-19 patients infected by Omicron variant with convulsion. [file 12879_2023_8556_MOESM3_ESM.doc]

**S3 Table** The biochemical and lymphocyte subsets examination of COVID-19 patients infected by Omicron variant with convulsion

| Parameters | Convulsion I group  (n=63) | Convulsion II group  (n=39) | *P* |
| --- | --- | --- | --- |
| Albumin(g/L) | 44.1±2.0 | 44.4±2.4 | 0.463 |
| Globulin(g/L) | 23.5(21.2-26.2) | 22.4(20.1-24.1) | 0.029 |
| ALT(U/L) | 20.2(15.5-24.6) | 21.6(16.3-27.0) | 0.377 |
| AST(U/L) | 52.5±15.7 | 56.4±20.1 | 0.290 |
| ALP(U/L) | 214.6±57.0 | 241.7±64.2 | 0.038 |
| LDH(U/L) | 353.7±84.7 | 366.3±80.2 | 0.464 |
| C3(g/L) | 1.0±0.2 | 1.0±0.2 | 0.114 |
| C4(g/L) | 0.3±0.1 | 0.3±0.1 | 0.505 |
| IgA(g/L) | 0.53(0.25-0.9) | 0.3(0.2-0.7) | 0.020 |
| IgG(g/L) | 6.7(5.8-9.2) | 6.5(5.2-7.7) | 0.068 |
| IgM(g/L) | 1.1±0.4 | 0.9±0.4 | 0.085 |
| Ca2+(mmol/L) | 1.0±0.2 | 1.1±0.1 | 0.586 |
| Lactic acid(mmol/L) | 1.8(1.4-2.6) | 2.2(1.6-3.2) | 0.108 |
| D-Dimer | 570(390-830) | 640(390-817) | 0.491 |
| lymphocyte subsets(/ul) | 1736(1129-3140) | 1883(1069-2855) | 0.929 |
| CD3^+^ Tcell count(/ul) | 1046(630-1962) | 1091(497-1901) | 0.692 |
| CD3^+^CD4^+^ T cell count(/ul) | 547(351-1154) | 611(296-1201) | 0.715 |
| CD3^+^CD8^+^ T cell count(/ul) | 349(206-666) | 353(190-506) | 0.603 |
| CD3^-^CD19^+^ B cell count(/ul) | 441(198-761) | 406(187-734) | 0.885 |
| NK cell count(/ul) | 194(114-312) | 191(86-341) | 0.692 |

Notes: The data presented as median [interquartile range], mean± standard deviation and n (%). The univariate analyses were performed using Mann-Whitney U-test for skewed distribution variables, t-test for normal distribution variables and the chi-square test for categorical variables. Abbreviation: ALT: alanine transaminase, AST:aspartate transaminase, ALP: alkaline phosphatase, LDH:lactate dehydrogenase. *P*<0.05 had statistical significance.
